# Spatiotemporal patterns and social determinants of county life expectancy in the Yangtze River Economic Belt

**DOI:** 10.3389/fpubh.2025.1521414

**Published:** 2025-04-25

**Authors:** Youming Dong, Mengcheng Wang, Yaya Song, Zeyu Yi, Jiulang Peng, Xiyan Mao, Xianjin Huang

**Affiliations:** ^1^School of Geography and Ocean Science, Nanjing University, Nanjing, China; ^2^Key Laboratory of Carbon Neutrality and Territorial Space Optimization, Ministry of Natural Resources, Nanjing, China

**Keywords:** life expectancy, spatiotemporal pattern, social determinants of health, geographically and temporally weighted regression model, the Yangtze River Economic Belt

## Abstract

**Background:**

Revealing the spatiotemporal differentiation characteristics of population life expectancy (LE) and exploring the spatiotemporal heterogeneity in impacts of social determinants of health (SDOH) is a crucial foundation for the scientific allocation of regional public resources and the formulation and implementation of localized public health policies.

**Materials and methods:**

The study focused on 1,068 county-level units in the Yangtze River Economic Belt (YREB) of China, utilizing census data from 2000, 2010, and 2020 to uncover the spatiotemporal differentiation patterns of county-level LE. The Geographically and Temporally Weighted Regression (GTWR) model was employed to analyze the spatiotemporal heterogeneity in impacts of various SDOH on LE and the differences in effects among different types of county-level administrative divisions.

**Results:**

(1) From 2000 to 2020, the average LE in the counties of the YREB had gradually increased from 72.3 years to 81.3 years, with a spatial pattern of LE showing that the eastern region exceeded the central region, which exceeded the western region. (2) The high-high clusters were primarily concentrated in urban agglomerations, while low-low clusters were predominantly located in the western region of the YREB. (3) Overall, the gender ratio (GR) and family size (FS) negatively impacted LE, while the average years of education (AYE), the logarithm of per capita disposable income [PDI(ln)], per capita housing area (PHA), and healthcare professionals per 1,000 people (PHP) had positive effects. (4) The impact of different SDOH varied across space and time. Furthermore, the effects of different SDOH varied among different types of county-level administrative divisions.

**Conclusion:**

These findings encourage local policymakers to focus on socioeconomic development at the county level, rationally allocate public resources, and formulate and implement localized public health policies in a tailored and orderly manner, thereby promoting spatial equity in population health.

## 1 Introduction

Life expectancy (LE) can comprehensively reflect the economic and social development as well as the quality of life of a country or region (1, 2). It has been widely used to measure geographical disparities in public health (3–5). However, since the 21st century, spatial inequalities in LE between regions and between rural and urban areas have become increasingly pronounced (3, 6, 7), particularly in some developing countries (8, 9), posing significant challenges to social equity and sustainable development. In this context, understanding the spatial characteristics and trends of LE and identifying its key influencing factors are essential for promoting spatial equity in LE.

Social determinants of health (SDOH) are considered major factors contributing to geographical variations in LE (5, 8, 10). They are the conditions in the environments where people are born, grow, live, learn, work, play, worship, and age, affecting their health outcomes (11). SDOH encompasses a wide range of factors, including race/ethnicity (11), economic status (12), occupation (13), education (11), housing (2, 14), healthcare access (15, 16), etc. As SDOH are rooted in local development and significantly influence public health policy (11), understanding the relationship between SDOH and LE is essential for the rational allocation of public health resources and the formulation of scientifically local development policies aimed at improving population health. This has important practical implications for enhancing well-being and promoting sustainable regional development.

It is essential to explore the relationship between SDOH and LE from the perspective of spatiotemporal variation. Due to differences in social environments and economic development across regions, SDOH may exhibit significant geographical variation, influencing the spatial equilibrium and synchronicity of changes in health outcomes. A deeper understanding of the spatiotemporal patterns of this relationship is crucial for developing local public health policies tailored to regional conditions. However, existing studies have predominantly used traditional statistical models (7, 10, 17) to reveal the overall impact of SDOH on LE or spatial econometric models to examine the spatial effects of SDOH on LE (8, 18). With the advancement of geographic spatial models, scholars have applied the Geographic Weighted Regression (GWR) model to reveal the spatial heterogeneity in the impact of SDOH on LE (19, 20). However, this model does not account for temporal variations. The Geographically and Temporally Weighted Regression (GTWR) model extends GWR by considering the spatiotemporal non-stationarity of the data. It can reveal how the regression coefficients of independent variables change across different spatial and temporal locations (21). However, existing studies on the impact of SDOH on LE have rarely utilized this model. Therefore, this paper introduced the GTWR model to investigate the spatiotemporal heterogeneity of impact of SDOH on LE.

Additionally, in terms of spatial scale, existing studies on the spatiotemporal dynamics of LE and the impact of SDOH have mainly focused on national levels, internal administrative units, and census districts. These studies have explored the subject globally, as well as in different countries and regions (7, 10, 13, 18, 22, 23). In China, most relevant studies have focused on the provincial and city levels. These studies have found that LE in eastern provinces is generally higher than in central and western provinces. And provincial capitals and eastern coastal cities tend to have higher LE compared to cities on the provincial periphery or at provincial borders. Based on these findings, the impact of socioeconomic factors such as population structure, economic development, education, and healthcare facilities has been explored at the provincial and city scales (8, 9, 19, 24). Counties serve as important units in China that connect urban and rural areas. They are also the foundational spaces for developing the socio-economy, implementing public health policies, and ensuring the well-being of the population (25). This paper argues that the research at the county level can reveal health disparities in a more nuanced and profound manner compared to studies at the provincial or municipal levels. It also helps illustrate the urban–rural divide in China. Furthermore, revealing the health effects of income, education, housing, and healthcare services at the county level can enhance the optimization of public resource allocation and promote socioeconomic development in these areas. However, due to limitations such as incomplete statistical data at the county level in China, research on LE assessment at the county level and the impact of social determinants remains relatively scarce.

The Yangtze River Economic Belt (YREB) is a key area for population concentration and high-quality development in China. Improving population health and promoting spatial equity in the region is essential for achieving the “Healthy China 2030” goal (26) and facilitating high-quality development in the YREB. However, long-standing imbalances in population distribution and public resource allocation (27), along with substantial disparities in economic development (28), may have contributed to significant spatial inequalities in population health. Therefore, it is crucial to examine the YREB to uncover the spatiotemporal characteristics of LE and the spatiotemporal effects of SDOH, providing a foundation for the development of regionally tailored public health policies.

Building on the research background and existing literature, this study focused on the YREB at the county level to examine the spatiotemporal dynamics of LE from 2000 to 2020 and analyze the impact of SDOH on LE from the perspective of spatiotemporal heterogeneity. The study comprised three main aspects. First, it identified the spatiotemporal patterns of county-level LE from 2000 to 2020 by constructing life tables. Second, it assessed the spatiotemporal heterogeneity of SDOH effects on LE using the GTWR model. Finally, it examined differences in the influence of SDOH on LE across various county administrative divisions to reveal urban–rural disparities.

This study makes several contributions to the existing literature. First, it focuses on the YREB in China, a key cross-regional national strategic development zone, and explores the spatiotemporal differentiation of LE at the county level, thereby enriching both the research area and spatial scale in research on the spatiotemporal dynamics of LE. Second, the paper investigates the impact of SDOH on LE from the perspective of spatiotemporal heterogeneity, addressing the gap in the existing literature regarding temporal heterogeneity. Additionally, the study highlights county-level urban–rural differences by analyzing the relationship between SDOH and LE across various county administrative divisions, a perspective less explored in existing research on SDOH at the county level in China. The findings contribute to a deeper understanding of health disparities between urban and rural areas in China and provide valuable insights for public health policymaking at the county level.

The remainder of this paper is structured as follows. Section 2 presents the materials and methods. Section 3 reports the results. Sections 4 and 5 provide the discussion and conclusions, respectively.

## 2 Materials and methods

### 2.1 Study area

**Figure 1:**
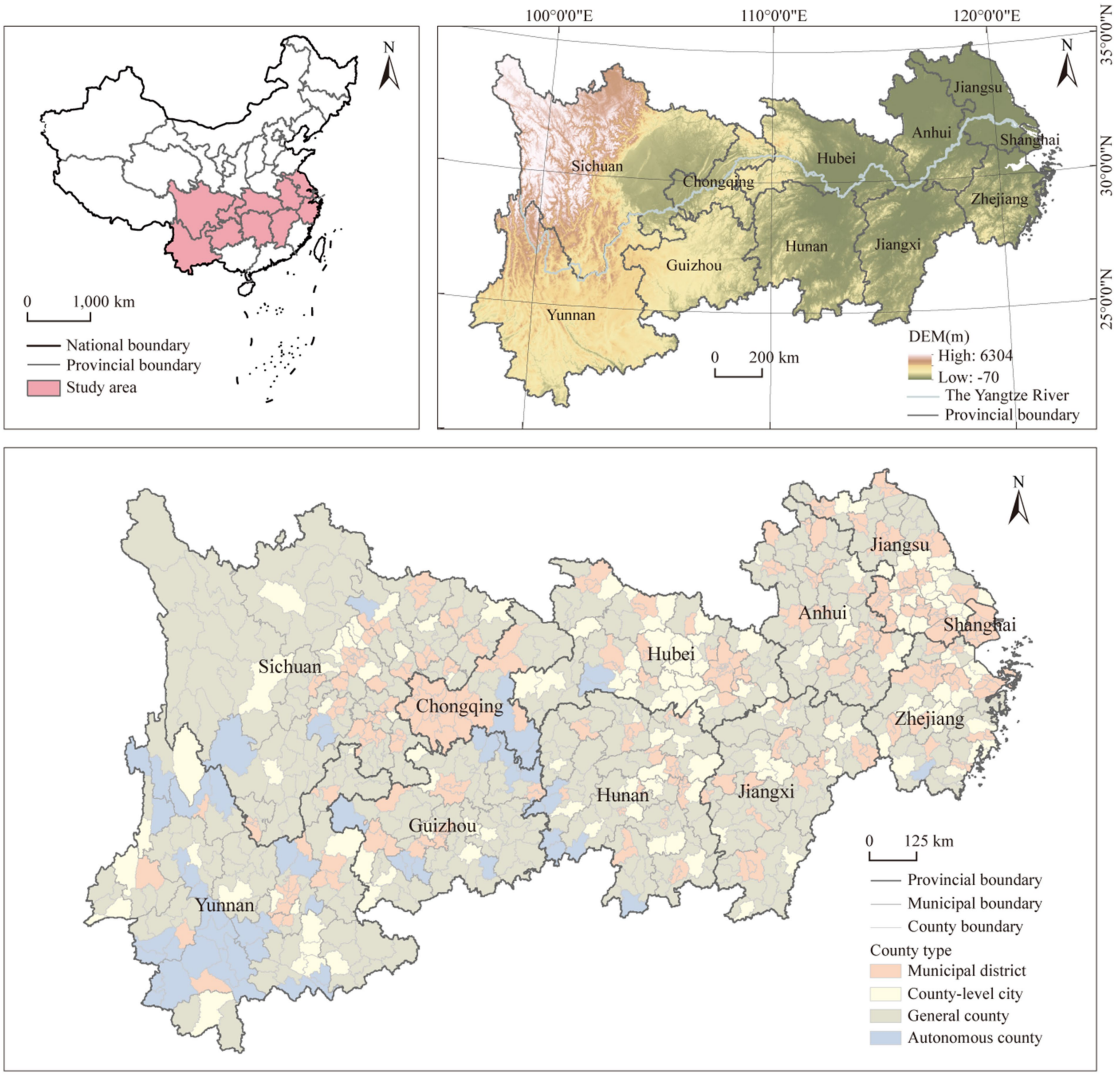
The study area.

The YREB spans the eastern, central, and western regions of China, encompassing 11 provinces (municipalities), including Shanghai, Jiangsu, Zhejiang, Anhui, Jiangxi, Hubei, Hunan, Chongqing, Guizhou, Sichuan, and Yunnan (Figure 1). It covers approximately 21.4% of the land area of China. By 2020, this region had accounted for approximately 43.0% of China’s population and 46.6% of its total GDP (29), making it a crucial area for socioeconomic development in China. The study area comprises 1,068 counties in the YREB, encompassing four types of administrative divisions: municipal districts, county-level cities, general counties, and autonomous counties, which partially reflect the urban–rural gradient. Additionally, the YREB is divided into eastern region (Shanghai, Jiangsu, Zhejiang, Anhui), central region (Jiangxi, Hubei, Hunan), and western region (Chongqing, Sichuan, Guizhou, Yunnan) for comparative analysis.

### 2.2 SDOH affecting LE

#### 2.2.1 Selection of SDOH

Due to the broad and ambiguous definition of SDOH and the variability of data across different countries and regions, there is currently no clear and unified indicator system for SDOH, and selection should be based on the specific data of each country and region. Existing studies in China have primarily focused on the impact of SDOH such as income, education, housing, healthcare services, etc. (9, 14, 19, 30, 31). Based on a systematic literature review, Wang et al. (8) proposed an analytical framework for SDOH at the provincial scale in China, encompassing three dimensions: socioeconomic development and equity, healthcare resources, and population characteristics. This paper argued that the SDOH influencing LE at the county level can be extended to the provincial scale. Drawing on related studies and considering the availability of data from Chinese counties, this paper characterized SDOH in three dimensions: population characteristics, socioeconomic factors, and healthcare services.

Population characteristics generally include gender, ethnicity/race, family structure, etc., mainly reflecting social demographic composition and family upbringing environment, which may have positive or negative effects on LE (8, 11, 18). Since the majority of the population in YREB is Han Chinese, the study selected gender ratio (GR) and family size (FS) to reflect population characteristics at the county level.

Socioeconomic factors such as education, income, and housing can indicate individuals’ social status and economic wealth, which are significant determinants of LE (11, 14, 31). Education not only enhances the health literacy of individuals but also promotes employment and income growth (11). Moreover, higher income and homeownership can improve quality of life, thereby enhancing health (8, 14). Therefore, the study used average years of education (AYE), per capita disposable income (PDI), and per capita housing area (PHA) to reflect socioeconomic factors at the county level.

Healthcare services, as public health resources provided by the government, may exhibit spatially uneven distribution, potentially leading to geographical disparities in LE (11). Considering data availability, the study primarily used healthcare professionals per thousand people (PHP) to reflect healthcare services at the county level.

Additionally, we considered the impact of geographical environment on LE (32), which is included in the analysis as an additional factor. It is essential to note that, since the YREB is located in a subtropical zone with abundant and relatively uniform precipitation, and its temperature variation is mainly influenced by altitude, the study solely utilized altitude (ALT) to represent the geographical environment.

In summary, this paper selected six social determinants—GR, FS, AYE, PDI, PHA, and PHP—and one environmental factor, ALT (Table 1).

**Table 1:** SDOH of LE at the county level.

Dimension	Factor	Unit	Description or definition	2000		2010		2020	
				Mean	S.D.	Mean	S.D.	Mean	S.D.
Population characteristics	Gender ratio (GR)	%	The ratio of the male population to the female population in a region	107.59	5.08	105.26	4.73	105.27	4.90
	Family size (FS)	Person per household	The average number of people per household in a region	3.49	0.45	3.18	0.49	2.69	0.32
Socioeconomic factors	Average years of education (AYE)	Year	The average number of years of formal education (including adult education, excluding various training programs) received by the population aged 6 and above in a region	7.14	1.26	8.36	1.26	9.30	1.37
	Per capita disposable income (PDI)	Yuan	The total disposable income of residents in a region divided by the resident population	4055.18	2597.67	12876.54	7907.44	31661.66	13575.96
	Per capita housing area (PHA)	m^2^	The average residential building area owned by the population in a region	25.09	7.26	34.01	7.98	46.47	9.34
Healthcare services	Healthcare professionals per 1,000 people (PHP)	Person	The number of healthcare professionals owned per 1,000 people in a region	2.81	1.46	4.99	2.58	7.11	3.61
Supplementary factor	Altitude (ALT)	m	The average altitude in a region	685.25	879.16	685.25	879.16	685.25	879.16

The population size in the table refers to the resident population of a region.

#### 2.2.2 Descriptive statistics of variables

Table 1 presents the statistical descriptions of various social determinants. Regarding population characteristics, the average GR in counties during the study period was greater than 1, indicating a higher male population than female. The mean FS was approximately 3, suggesting an average household size of three people, with a decreasing trend over time.

In terms of socioeconomic factors, the average values of AYE, PDI, and PHA in counties increased significantly from 2000 to 2020, reflecting notable improvements in residents’ education levels, per capita income, and per capita housing space, which indicates an overall enhancement in quality of life. However, the standard deviations of these three variables increased over time, suggesting widening disparities in education, income, and housing across counties.

For healthcare services, both the mean and standard deviation of county PHP increased from 2000 to 2020, demonstrating an overall improvement in healthcare services. However, this also indicates a growing inequality in healthcare resource distribution among counties.

### 2.3 Methods

#### 2.3.1 Abbreviated life table

LE generally refers to the life expectancy at birth, which is the number of years a cohort of individuals born simultaneously can expect to live if mortality rates remain constant across all age groups (2). This study constructed abbreviated life tables to estimate LE based on the population and mortality data for each age group at the county level. The detailed calculation steps and formulas (Equations 1–7) of the life table can be found in (33).

First, the age-specific mortality rate *m_x_* for each age group in the county was calculated as follows:


(1) $$m_{x}=b_{x}/a_{x}$$

Where *m_x_* denotes the mortality rate for age group *x* (*x* = 0 years, 1–4 years, 5–9 years, …) in the county. *a_x_* represents the total population of age group *x* in the county, and *b_x_* represents the number of deaths in age group *x* in the county.

Second, the probability of death *q_x_* was calculated for each age group *x* in the county:


(2) $$q_{x}=\frac{2m_{x}}{2+m_{x}}$$

Where *q_x_* represents the likelihood that an individual who survives to exact age *x* will die within the following year.

Third, the number of survivors *l*_0_ at age 0 is typically set at 100,000, and the number of tabulated deaths *d_x_* and survivors *l_x_* were calculated for each age group *x* in the county:


(3) $$d_{x}=l_{x}*q_{x}$$

(4) $$l_{x+1}=l_{x}-d_{x}$$

Where *d_x_* represents the number of deaths (not the actual number of deaths) in age group *x* in the life table. *l*_*x +* 1_ represents the number of survivors in age group *x* minus the number of deaths in age group *x*, i.e., the number of individuals who survive to the exact age *x +* 1.

Fourth, the average person-years lived *L_x_* and the cumulative person-years lived *T_x_* were calculated for each age group *x*:


(5) $$L_{x}=\frac{l_{x}+l_{x+1}}{2}$$

(6) $$T_{x}=\sum L_{x}$$

Where the average person-years lived *L_x_* represents the total number of years lived by a cohort of individuals (*l_x_*) who have reached a specific exact age *x* before progressing to age *x* + 1. The cumulative person-years lived *T_x_* is the sum of *L_x_* across all subsequent age groups, representing the total remaining years that a cohort of individuals (*l_x_*) at a given exact age *x* is expected to live under the prevailing mortality conditions.

Finally, the average life expectancy *e_x_* was calculated:


(7) $$e_{x}=\frac{T_{x}}{l_{x}}$$

Based on the calculation results, the average life expectancy of the 0-year age group was used as the final estimate of LE.

#### 2.3.2 Spatial autocorrelation

To uncover the spatial correlation characteristics of county LE in the YREB from 2000 to 2020, we initially utilized the Global Moran’s *I* to explore the overall spatial correlation. Then, we employed local indicators of spatial association (LISA) to further uncover five spatial correlation patterns, including high-high cluster, low-low cluster, high-low outlier, low-high outlier, and no significant (34). The basic formula (Equation 8) for Global Moran’s *I* is as follows:


(8) $$I=\frac{n\sum_{i=1}^{n}\sum_{j=1}^{n}w_{ij}\left(x_{i}-\bar{x}\right)\left(x_{j}-\bar{x}\right)}{\sum_{i=1}^{n}\sum_{j=1}^{n}w_{ij}\sum_{i=1}^{n}{\left(x_{i}-\bar{x}\right)}^{2}}\left(i\neq j\right)$$

The calculation formula (Equation 9) for LISA is as follows:


(9) $$I_{i}=\frac{\left(x_{i}-\bar{x}\right)\sum_{j=1}^{n}w_{ij}\left(x_{i}-\bar{x}\right)}{\frac{1}{n}\sum_{i=1}^{n}{\left(x_{i}-\bar{x}\right)}^{2}}\left(i\neq j\right)$$

Where *I* represents the value of the global Moran index. *I_i_* is the value of the local Moran index. *n* represents the number of counties in the YREB. *x_i_* and *x_j_* denote the LE values of county *i* and county *j*, respectively. $\bar{x}$ stands for the average LE of all counties in the YREB. *w_ij_* denotes spatial weights. The positive values of Global Moran’s *I* indicate spatial agglomeration, with larger values suggesting stronger spatial agglomeration. While negative values mean spatial heterogeneity, with smaller values indicating stronger spatial heterogeneity. The value of 0 indicates no spatial autocorrelation.

#### 2.3.3 The GTWR model

The GTWR model is a geospatial analytical tool that integrates both spatial and temporal dimensions, aiming to uncover the dynamics of variable relationships across these scales (21). The model estimates regression coefficients for an observation by assigning local weights based on its geographical location and time. Compared to the GWR model, the GTWR model is better suited for analyzing complex spatiotemporal data, providing more accurate results. In this study, the GTWR model was primarily used to analyze the impact of SDOH on LE. Prior to conducting the GTWR analysis, scatter plots were used to initially explore the relationship between independent and dependent variables, followed by standardization, a multicollinearity test, and a spatiotemporal non-stationarity test.

First, the relationships between different SDOH and LE were initially assessed using scatterplots. The scatter plots indicated a potential non-linear relationship between PDI and LE. To ensure that PDI and other explanatory variables were included in the same model, this study followed the approach of previous studies by taking the natural logarithm of PDI (PDI(ln)) and including it (12, 17), along with other explanatory variables, in the model.

Second, the variables were standardized, and multicollinearity was tested. Standardization ensures model robustness and comparability of regression coefficients, while the multicollinearity test ensures the model’s credibility. Diagnostic results showed that the VIF for the seven explanatory variables was less than 7.5, indicating independence among the variables. Thus, all seven explanatory variables were included in the GTWR model for analysis.

Additionally, the spatiotemporal non-stationarity of the sample was tested. Previous research has indicated that comparing the interquartile range of the GTWR model with twice the standard errors of the OLS model is an effective testing method (35, 36). Table 2 demonstrates that all variables exhibit significant local variation, indicating the presence of spatiotemporal non-stationarity. Therefore, the impact of spatiotemporal heterogeneity of the variables was analyzed using the GTWR model.

**Table 2:** Spatiotemporal non-stationarity test of the GTWR model.

Variable	Interquartile (GTWR)	2*SE (OLS)	Extra local variation
Intercept	0.170	0.030	Yes
GR	0.170	0.036	Yes
FS	0.120	0.032	Yes
AYE	0.140	0.030	Yes
PDI (ln)	0.130	0.022	Yes
PHA	0.070	0.022	Yes
PHP	0.210	0.028	Yes
ALT	0.180	0.012	Yes

Based on the above data tests, GR, FS, AYE, PDI(ln), PHA, PHP, ALT, and LE were included in the analysis using the GTWR model. The GTWR model analysis was performed using the GTWR plug-in in ArcGIS 10.7. The basic equation formula (Equation 10) of the GTWR model is as follows:


(10) $$\begin{array}{l} y_{i}=\beta_{0}\left(\mu_{i},v_{i},t_{i}\right)+\beta_{1}\left(\mu_{i},v_{i},t_{i}\right)*GR_{i}+\beta_{2}\left(\mu_{i},v_{i},t_{i}\right)*FS_{i}+\\ \beta_{3}\left(\mu_{i},v_{i},t_{i}\right)*AYE_{i}+\beta_{4}\left(\mu_{i},v_{i},t_{i}\right)*PDI{\left(\ln\right)}_{i}+\\ \beta_{5}\left(\mu_{i},v_{i},t_{i}\right)*PHA_{i}+\beta_{6}\left(\mu_{i},v_{i},t_{i}\right)*PHP_{i}+\\ \beta_{7}\left(\mu_{i},v_{i},t_{i}\right)*ALT_{i}+\varepsilon_{i} \end{array}$$

Where *y_i_* represents the LE of county *i. β_0_*(*μ_i_, ν_i_, t_i_*) denotes the regression intercept term for the county *i*. (*μ_i_, ν_i_, t_i_*) represents the spatiotemporal location of the county *i*. *GR*_i_, *FS*_i_, *AYE*_i_, *PDI(ln)*_i_, *PHA*_i_, *PHP*_i_, and *ALT*_i_ represent the observed values of the independent variables for county *i* at the spatiotemporal point (*μ_i_, ν_i_, t_i_*). *β_1_-β_7_* are the regression coefficients of SDOH for county *i* at the spatiotemporal point (*μ_i_, ν_i_, t_i_*). *ε_i_* denotes the residual of the county *i*.

In addition, to validate the superior fit of the GTWR model, this paper compared its parameters with those of the OLS, GWR, and TWR models. Table 3 shows that the GTWR model achieves the highest *R*^2^, adjusted *R*^2^, and the smallest AIC and residual sum of squares (*RSS*) among all models, indicating the best fit to the data. The model’s adjusted *R*^2^ of 0.900 suggests that the included factors explain 90% of the spatial and temporal variation in county LE.

**Table 3:** Comparison of parameters for models of OLS, GWR, TWR, and GTWR.

Parameter	OLS	GWR	TWR	GTWR
Bandwidth	—	0.115	0.150	0.115
AIC	−9588.193	−10670.100	−9737.310	−10884.700
*R* ^2^	0.837	0.888	0.846	0.900
*R*^2^-Adjusted	0.837	0.888	0.846	0.900
Residual sum of squares	9.363	6.442	8.839	5.745

### 2.4 Data sources

Population counts and death numbers for each age group, along with data on GR, FS, AYE, and PHA, were obtained from the China population census by county for 2000, 2010, and 2020. Data on PDI and PHP were obtained from the statistical yearbooks of provinces and cities in the YREB, as well as the statistical bulletins on national economic and social development at the county level for 2000, 2010, and 2020. Altitude data were acquired from the Digital Elevation Model provided by the Resource and Environmental Science Data Platform of the Chinese Academy of Sciences,^1^ using image correction for data extraction. Additionally, missing data for certain counties and years were estimated using interpolation methods.

## 3 Results

### 3.1 Descriptive analysis of LE

**Figure 2:**
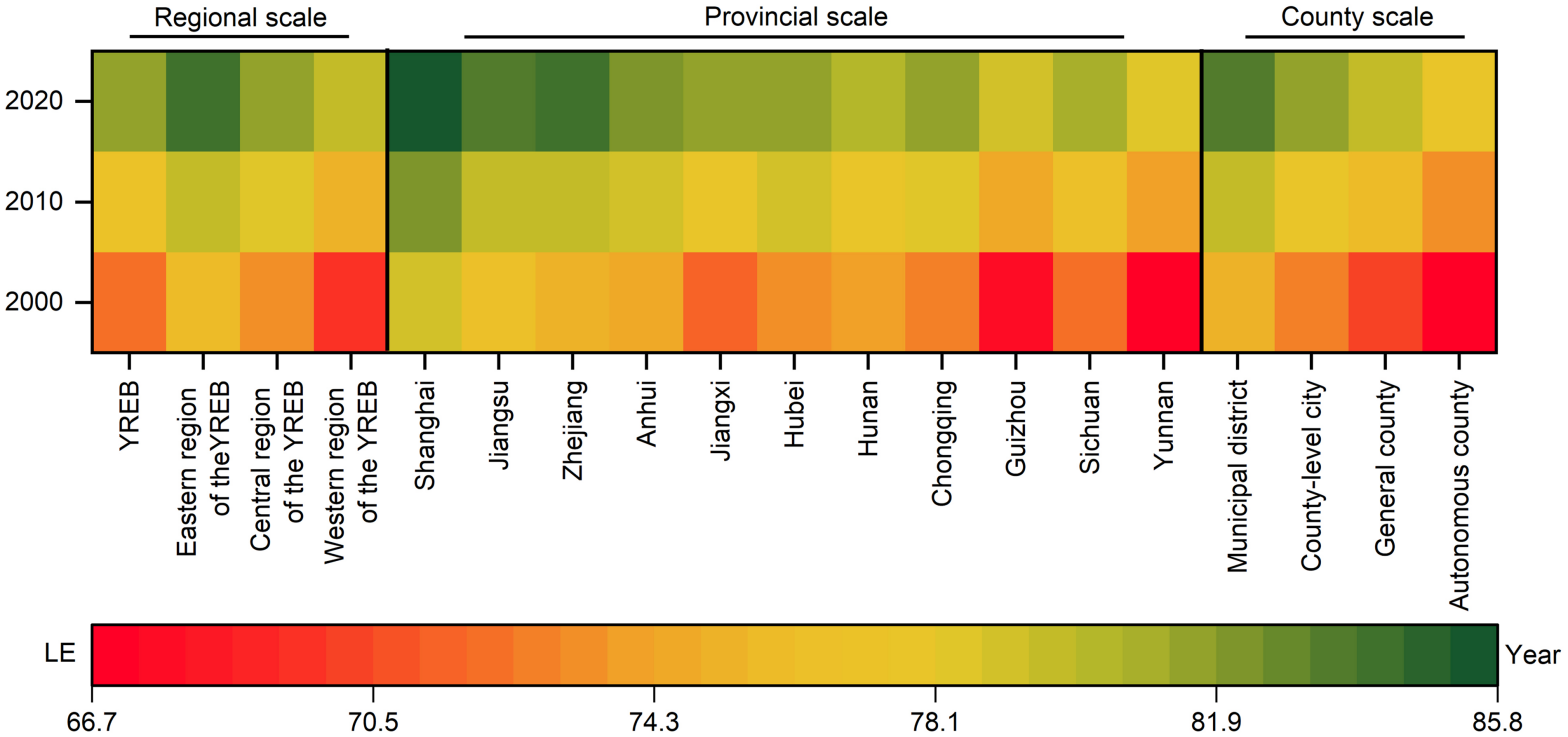
Statistical description of average LE in counties of the YREB, 2000—2020.

Figure 2 displays a heat map of average LE in counties at various spatial scales from 2000 to 2020. During this period, the average LE in the counties of the YREB had been increased from 72.3 years to 81.3 years, with a rise of 5.1 years from 2000 to 2010, and an increase of 3.9 years from 2010 to 2020. Regionally, the growth was highest in the western region (10.2 years), followed by the central region (8.3 years), and the eastern region (7.9 years). At the provincial level, significant increases were observed in Yunnan (11.2 years), Guizhou (11.0 years), and Jiangxi (10.8 years), while Shanghai (6.5 years) and Hunan (6.7 years) showed smaller increases. Among the four types of county-level administrative divisions, the increases ranked as autonomous counties (11.1 years) > general counties (9.5 years) > county-level cities (8.9 years) > municipal districts (8.1 years). Nevertheless, the average LE in counties at different spatial scales showed clear differentiation during the study period. Specifically, at the regional level, the average county LE was ranked as eastern > central > western. At the provincial level, Shanghai showed a relatively high LE, while Yunnan and Guizhou had comparatively lower values. At the county level, the order was municipal districts, followed by county-level cities, general counties, and then autonomous counties, in decreasing order.

### 3.2 Spatiotemporal patterns of LE

**Figure 3:**
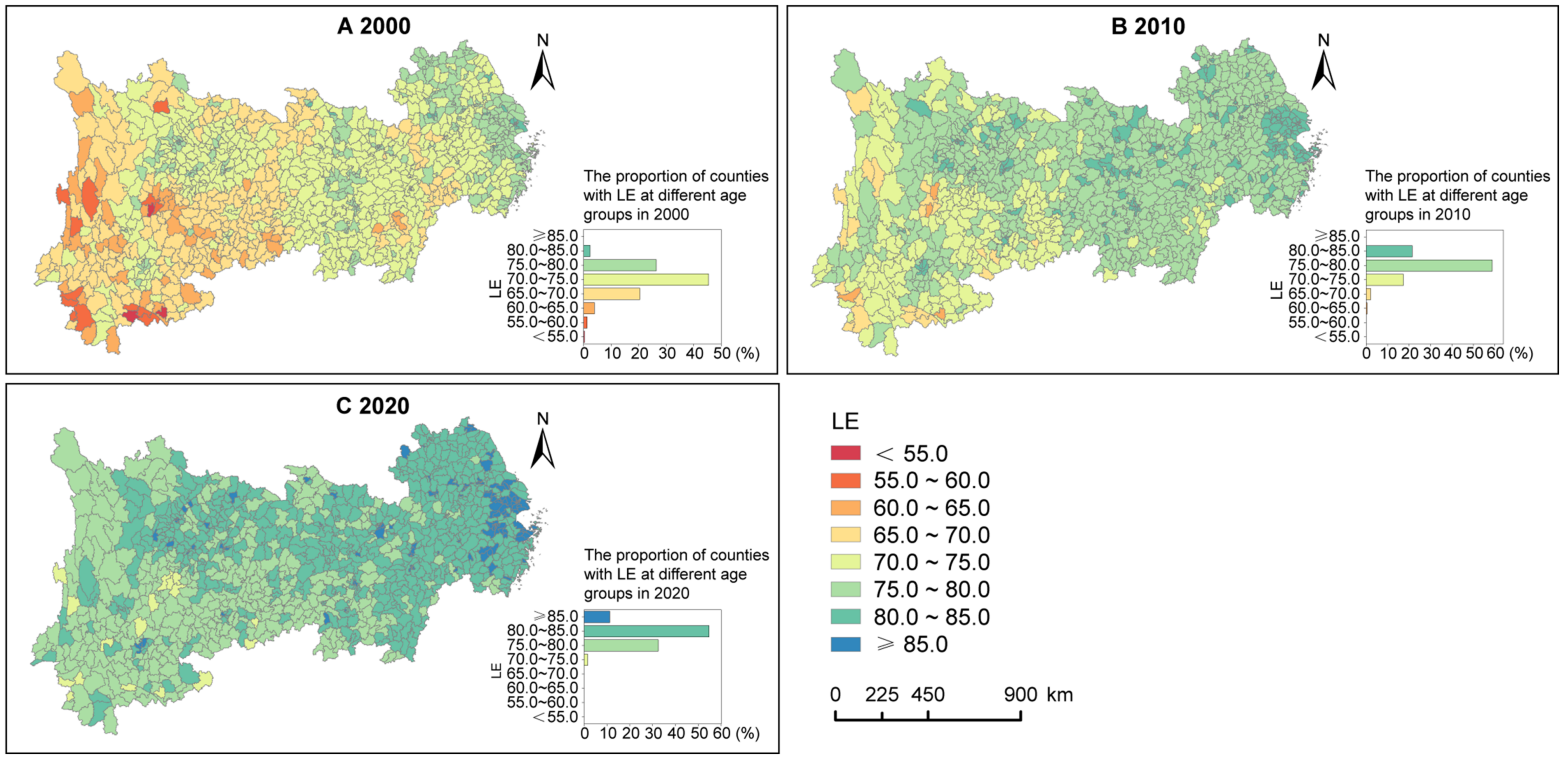
Spatiotemporal distribution of LE in counties of the YREB, 2000—2020. **(A)** 2000. **(B)** 2010. **(C)** 2020.

Using a 5-year interval, LE values for all counties in the YREB from 2000 to 2020 were divided into eight segments (Figure 3). During this period, LE in the counties of the YREB gradually decreased from east to west. Specifically, high-value counties were mainly located in the eastern coastal areas and around the provincial capitals and major cities of inland provinces, while low-value counties were predominantly found in the western region, particularly in Yunnan and Guizhou. Over time, the number of high-value counties in the YREB had been increased, while low-value counties had been decreased.

Moran’s *I* and Z scores (Table 4) indicate a moderate degree of spatial clustering in LE among the counties of the YREB, with the level of clustering initially weakening and then strengthening from 2000 to 2020.

**Table 4:** Global spatial autocorrelation results.

Year	Moran ‘*I*	Z (*I*)	*p*
2000	0.571	59.978	0.000
2010	0.518	54.387	0.000
2020	0.571	59.905	0.000

**Figure 4:**
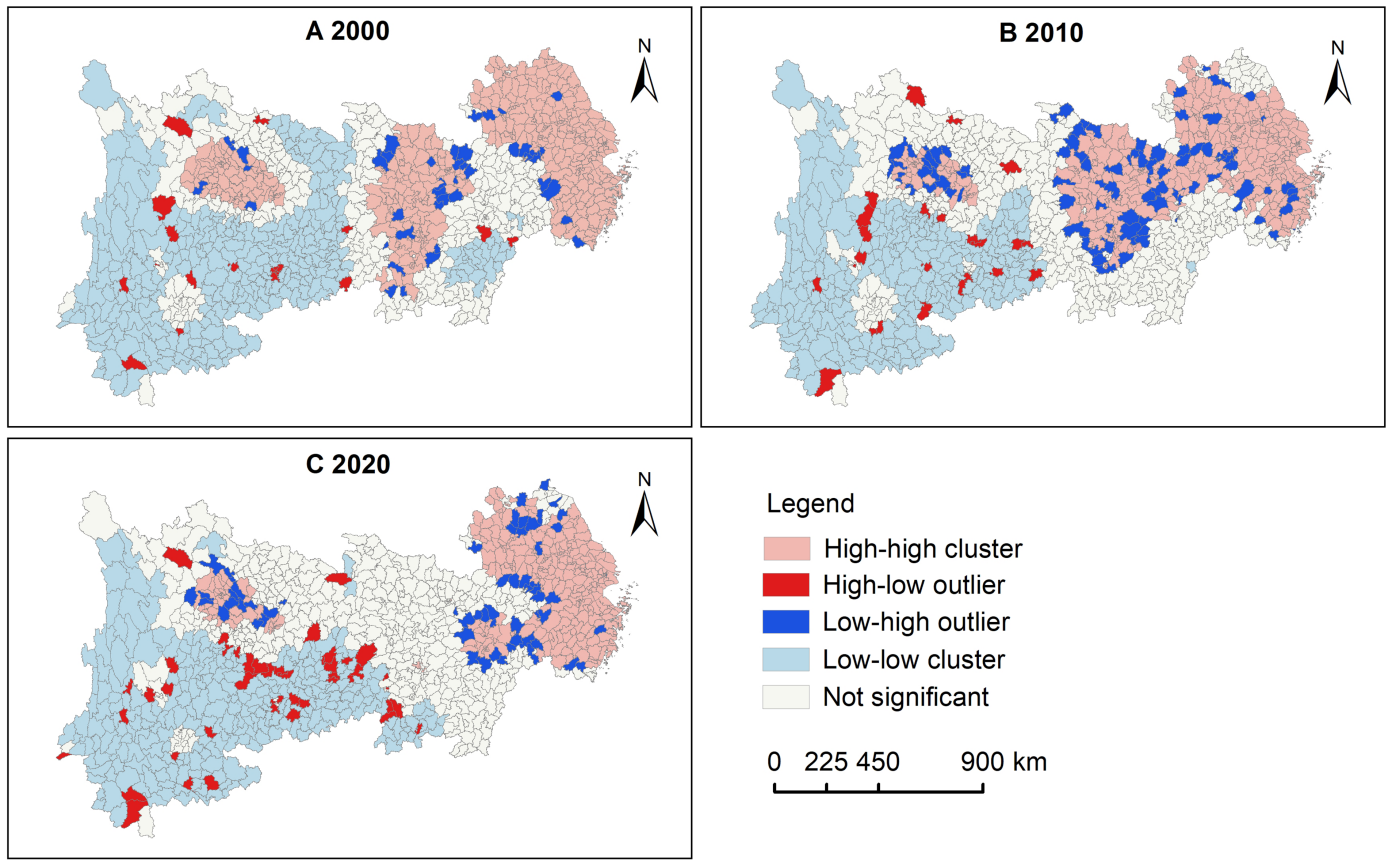
LISA maps of county LE in the YREB, 2000—2020. **(A)** 2000. **(B)** 2010. **(C)** 2020.

LISA maps (Figure 4) reveal a distinct “high in the east, low in the west” pattern of spatial clustering for LE. Specifically, high-high cluster areas were primarily located in the Yangtze River Delta, Chang-Zhu-Tan urban agglomeration, Cheng-Yu urban agglomeration, and the Wuhan metropolitan area and their surroundings in 2000. Over time, the high-high cluster areas in the Cheng-Yu urban agglomeration had been gradually shrunk, while those in the Wuhan metropolitan area and Chang-Zhu-Tan urban agglomeration had been nearly disappeared by 2020. In contrast, low-low cluster areas in 2000 were mainly found in Yunnan, Guizhou, Sichuan, Chongqing, and Jiangxi. As time progressed, the low-low cluster areas in Chongqing and Jiangxi had been gradually diminished and disappeared. Additionally, the number of counties with high-low outliers had been increased over time, indicating that LE has been gradually improved in more counties in the western region.

### 3.3 Impacts of SDOH on LE

#### 3.3.1 General results

Table 5 presents the statistical description of the regression coefficients of the SDOH based on the GTWR model. The mean results provide an indication of the overall influence of the factors. The mean results of regression coefficients show that the overall impacts of GR and FS were negative. Specifically, for every unit increased in GR and FS in the county in 2000, the average LE decreased by 0.098 and 0.126 years, respectively. The negative impacts of GR and FS diminished over time.

**Table 5:** Statistical description of regression coefficients of SDOH based on the GTWR model.

Variable	2000				2010				2020			
	Mean	Min	Max	S.D.	Mean	Min	Max	S.D.	Mean	Min	Max	S.D.
GR	−0.098	−0.308	0.189	0.114	−0.068	−0.289	0.103	0.086	−0.046	−0.264	0.174	0.117
FS	−0.126	−0.341	0.146	0.139	−0.073	−0.149	0.119	0.052	−0.034	−0.192	0.318	0.110
AYE	0.351	0.131	0.585	0.113	0.320	0.051	0.487	0.073	0.325	0.046	0.455	0.104
PDI (ln)	0.143	−0.188	0.383	0.138	0.196	0.004	0.315	0.066	0.139	0.036	0.345	0.066
PHA	0.059	−0.156	0.285	0.111	0.077	−0.022	0.253	0.049	0.034	−0.042	0.101	0.033
PHP	0.187	−0.101	0.981	0.255	0.128	−0.017	0.666	0.159	0.115	−0.062	0.669	0.185
ALT	−0.219	−0.761	0.097	0.223	−0.070	−0.298	0.083	0.100	−0.030	−0.177	0.055	0.047

**Figure 5:**
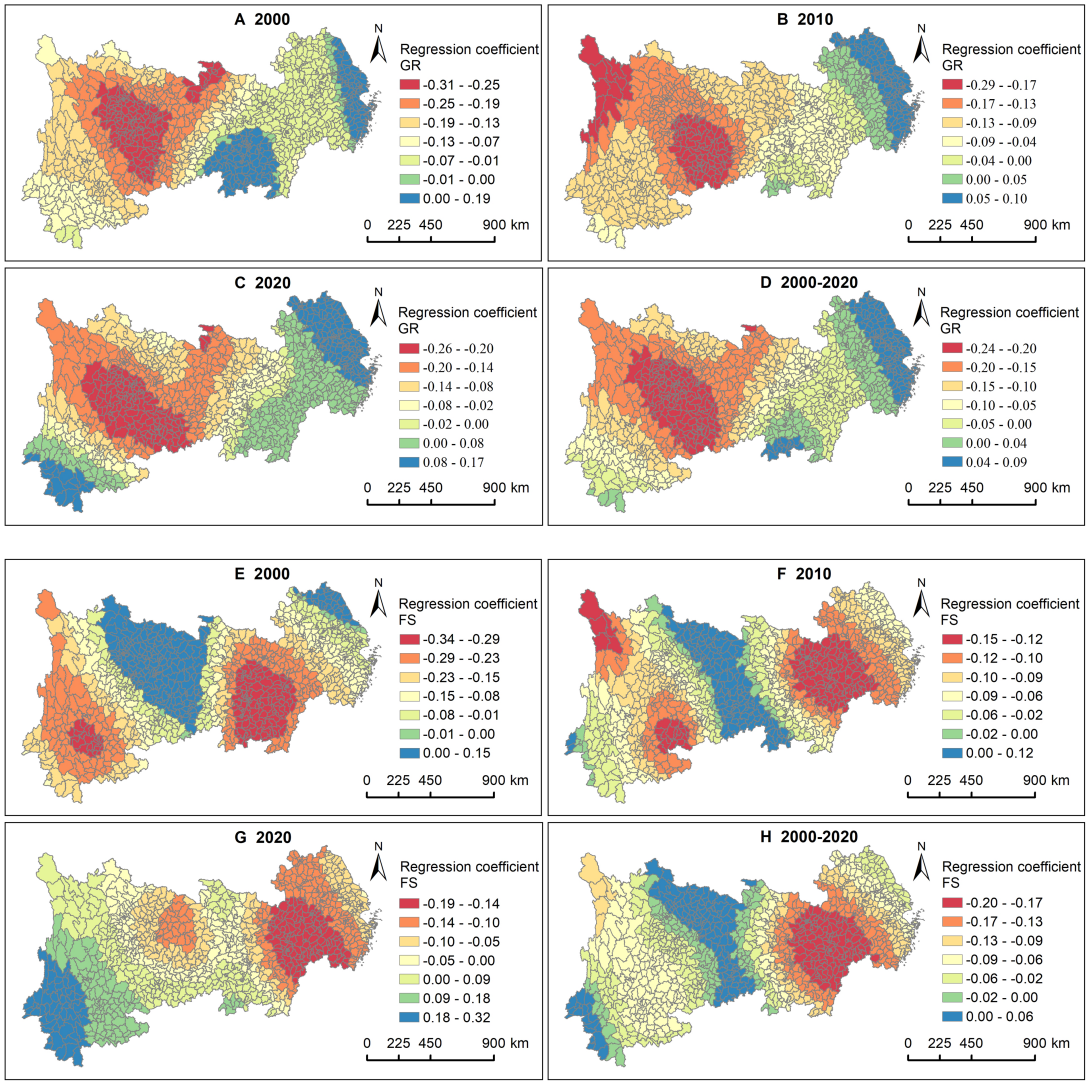
Association between LE and GR, FS. GR in **(A)** 2000, **(B)** 2010, **(C)** 2020, and **(D)** 2000—2020. FS in **(E)** 2000, **(F)** 2010, **(G)** 2020, and **(H)** 2000—2020.

**Figure 6:**
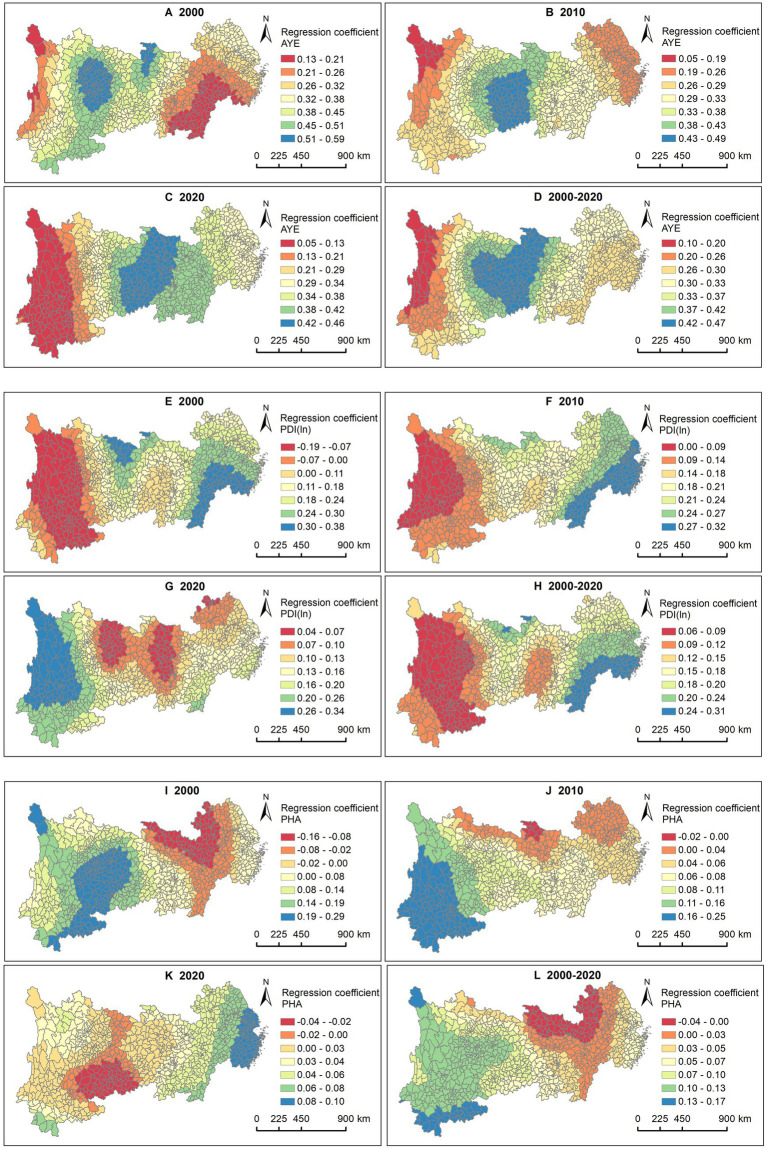
Association between LE and AYE, PDI (ln), PHA. AYE in **(A)** 2000, **(B)** 2010, **(C)** 2020, and **(D)** 2000—2020. PDI (ln) in **(E)** 2000, **(F)** 2010, **(G)** 2020, and **(H)** 2000—2020. PHA in **(I)** 2000, **(J)** 2010, **(K)** 2020, and **(L)** 2000—2020.

**Figure 7:**
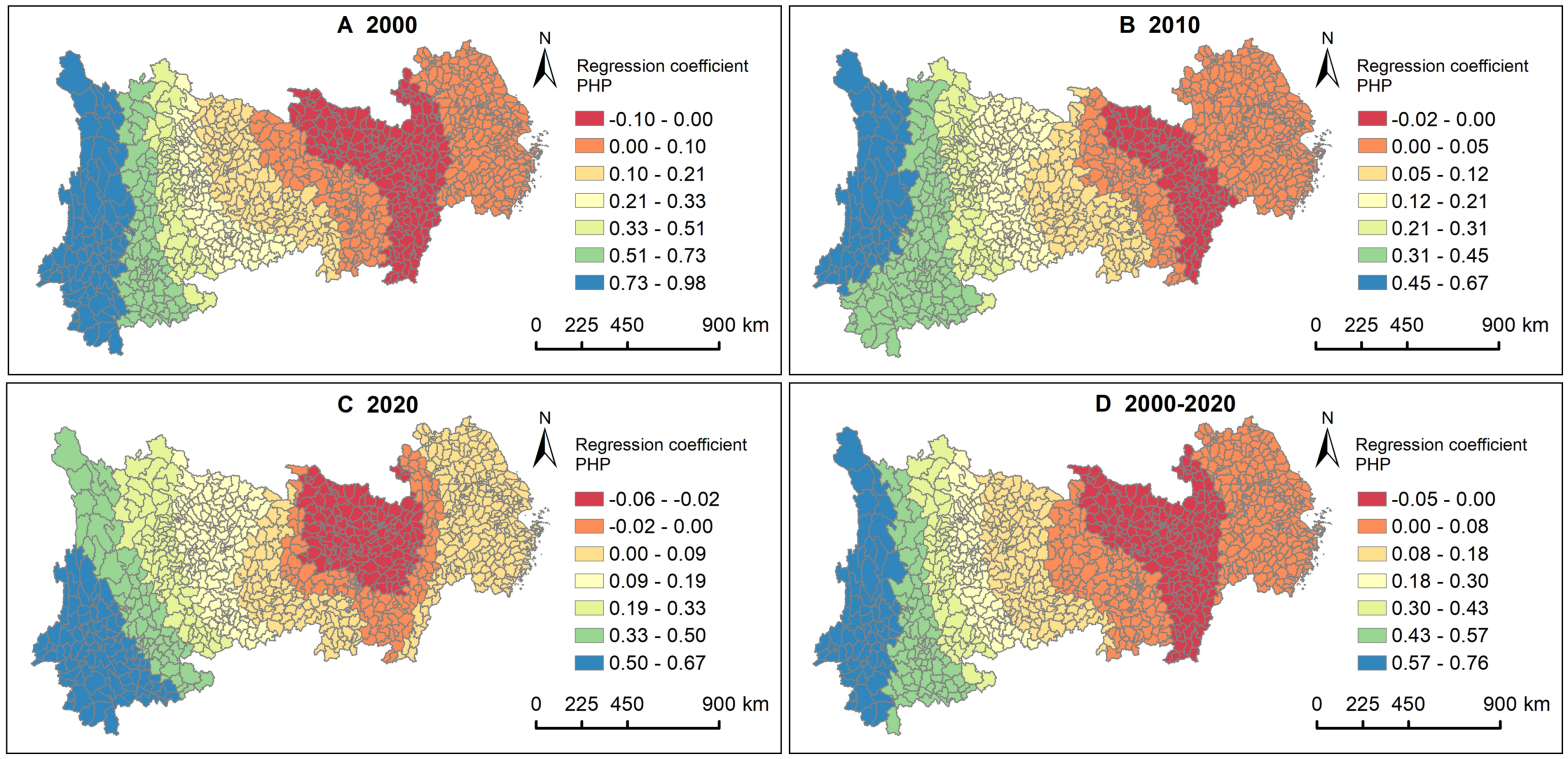
Association between LE and PHP. **(A)** 2000, **(B)** 2010, **(C)** 2020, **(D)** 2000—2020.

**Figure 8:**
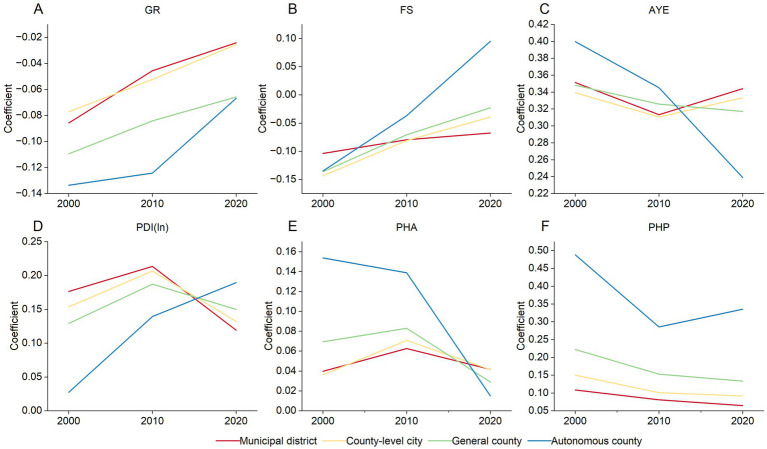
Temporal variation of the association between LE and SDOH across four county types, 2000—2020. **(A)** GR. **(B)** FS. **(C)** PDI (ln). **(D)** AYE. **(E)** PHA. **(F)** PHP.

The values in the table represent the average change in LE for each 1-unit change in the respective factor. This interpretation is the same as in Figures 5–8.

Regarding the socioeconomic factors, the mean regression coefficients of AYE, PDI(ln), and PHA generally indicate positive effects. Notably, AYE had a fully positive impact, with a mean regression coefficient greater than 0.30 across all years, demonstrating strong explanatory power among the social determinants. For every unit increased in AYE in the counties in 2000, LE, on average, improved by 0.351 years. Over time, the positive influence of AYE diminished on average.

The overall influences of PDI(ln) and PHA were relatively lower than that of AYE. For every unit increased in PDI(ln) and PHA in the counties in 2000, LE, on average, increased by 0.143 and 0.059 years, respectively. Over time, the positive impacts of PDI(ln) and PHA increased and then decreased.

Additionally, the impact of PHP was generally positive throughout the study period. For each unit increased in PHP in the county in 2000, LE, on average, increased by 0.187 years. The positive influence of PHP decreased over time.

#### 3.3.2 Spatiotemporal heterogeneity of impacts based on GTWR model

##### 3.3.2.1 Population characteristics

Regarding the GR, 80.34% of counties in the YREB exhibited negative impacts in 2000, particularly in the western region (Figures 5A–C). Over time, the number of counties with negative impacts had been decreased. In contrast, only 19.66% of counties had positive impacts in 2000, primarily concentrated in the eastern coastal areas, western Jiangxi, and southern Hunan. As time progressed, the counties with positive impacts had been expanded in the eastern and central regions. Concerning FS, counties with significant negative impacts were mainly located in the central region of the YREB and Yunnan in 2000 (Figures 5E–G). Over time, the counties with negative impacts had been gradually shifted eastward, concentrating along the eastern and central border by 2020. Conversely, counties with positive impacts in 2000 were mainly found in Jiangsu, Chongqing, Guizhou, and Sichuan, and the counties with positive impacts had been shifted westward gradually, predominantly appearing in Yunnan by 2020.

##### 3.3.2.2 Socioeconomic factors

Regarding the AYE, counties with significant positive impacts were primarily located in the western region of the YREB and western Hubei in 2000, with this influence gradually shifting eastward over time (Figures 6A–C). In 2020, it was mainly concentrated in the central region. In terms of PDI(ln), counties with significant positive impacts were predominantly found in Zhejiang, Shanghai, Jiangsu, Jiangxi, and Anhui in 2000 and 2010 (Figures 6E–G). However, this influence had been shifted to the western provinces of Sichuan and Yunnan by 2020. The spatiotemporal impact characteristics of PHA differed from those of income (Figures 6I–K). Specifically, counties with significant positive impacts were mainly in the western region of the YREB in 2000, focusing on Yunnan in 2010, but by 2020, this influence had moved to the eastern region.

##### 3.3.2.3 Healthcare services

The results for all 3 years show that PHP had a more significant positive impact in the western regions of Sichuan and Yunnan compared to other areas (Figures 7A–C). This may be closely related to the relatively underdeveloped healthcare conditions in the western region of China.

#### 3.3.3 Impacts among different county types

Figure 8 illustrates the changes in the mean regression coefficients of different SDOH across the four types of county-level administrative divisions from 2000 to 2020. In terms of demographic characteristics, the GR had a negative impact on LE across all four county types, with stronger effects in autonomous counties and general counties compared to municipal districts and county-level cities (Figure 8A). This partly reflected greater health disparities by gender in rural and ethnic minority areas. Over time, the negative impacts had been gradually diminished across all county types. FS generally had a predominantly negative impact (Figure 8B). As time progressed, this negative impact had been diminished to varying degrees across all four county types. In 2020, FS showed a positive impact in autonomous counties.

During the study period, socioeconomic factors demonstrated positive impacts across all four county types. The positive effects of AYE and PHA on LE in autonomous counties were more significant than in the other three county types in 2000 (Figures 8C,E). This result indirectly indicated the high demand for education and housing among ethnic minority residents in the early 21st century. However, this positive impact had been noticeably weakened in autonomous counties over time, while exhibiting fluctuations in the other three county types. In 2020, the positive effects of AYE and PHA on LE in autonomous counties were weaker than those in municipal districts, county-level cities, and general counties. In contrast, the positive impact of PDI(ln) on LE decreased in the order of municipal districts, county-level cities, general counties, and autonomous counties in 2000 (Figure 8D). Over time, this positive effect had been gradually increased in autonomous counties, while it rose and then fell in the other three county types.

Additionally, during the study period, the PHP exhibited a positive impact across all four county types, with the overall impact decreasing in the order of autonomous counties, general counties, county-level cities, and municipal districts (Figure 8F). This trend may be related to the lack of healthcare service resources in rural and ethnic minority areas. Throughout this period, the positive impact of PHP had been gradually diminished in general counties, county-level cities, and municipal districts, while showing a decrease followed by an increase in autonomous counties.

## 4 Discussion

### 4.1 Improvement and geographical inequality in LE

Overall, LE at the county level in the YREB had significantly increased during the study period, especially in the underdeveloped western regions, confirming previous findings (37). This result reflected the progress in improving the well-being of residents and promoting health equity in disadvantaged areas of China. In addition, the findings of this study revealed a generally higher level of LE compared to that of the Yellow River basin in China during the same period (38). This underscores the effectiveness of the regional policy promoting high-quality development in the YREB.

Despite significant improvements in county LE, a clear spatial divergence remains, with higher values in the eastern regions and lower values in the western regions (39). Additionally, we observe a sequential decline in the average LE across municipal districts, county-level cities, general counties, and autonomous counties, highlighting urban–rural health disparities in China (6). Moreover, counties with high and low LE during the study period exhibited a distinct “core-periphery” spatial structure within urban agglomerations and their peripheral areas, respectively. This spatial feature was also validated in the Beijing-Tianjin-Hebei region of China (20), indirectly suggesting the positive impact of urbanization on LE (40).

### 4.2 The heterogeneous impacts of SDOH

We found the overall effects of GR and FS on LE were negative during the study period, while AYE, PDI(ln), PHA, and PHP had primarily positive effects on LE. These findings are consistent with previous research (14, 19, 24, 41).

Regarding the spatiotemporal heterogeneity of effects in SDOH, we observed that during the study period, the GR was positively correlated with LE in eastern counties of the YREB, meaning that a higher proportion of males was associated with longer LE. Although existing studies have shown that females generally have higher LE than males (11), gender is not the sole social determinant of LE. One possible explanation is that men in the developed eastern region are more likely to improve their education and income levels, thereby narrowing the gap of LE with women. While this explanation has some scientific basis (42), the specific reasons require further verification. As for FS, the results showed that larger families tend to negatively impact LE, which differs from previous findings (24). This result may reflect the modernization of family structures in China (43). Under traditional values, large families are more common in rural or underdeveloped areas, where poverty may exacerbate the economic burden of large households, negatively affecting health (44). In contrast, smaller families are more prevalent in urban and developed regions of China, where proximity to quality healthcare and reduced economic pressure contribute to better health outcomes.

Regarding socioeconomic factors, education had the most significant positive impact on LE. The effect of education on LE during the study period was particularly pronounced in the central and western regions of the YREB, compared to the eastern region. These findings are consistent with results from previous studies at the provincial level in China (19). This indicates that education plays a crucial role in improving health outcomes. In terms of income, the significant positive effect shifted from the eastern to the western region during the study period. This may be due to the rapid economic growth in the eastern region in the early 21st century, which boosted income levels and improved health. However, as income continued to rise, the health benefits may have diminished, as evidenced by the marginal effect of income (45). In contrast, the western region, having experienced more policy support for economic development and employment in recent years, began to see more pronounced health benefits from income growth (46). Unlike the spatial distribution of effects in income, the significant positive impact of housing area shifted from the western to the eastern region during the study period. This could be attributed to the relatively poor housing conditions in the western region in the early 21st century, where improving housing conditions in underdeveloped areas had more substantial effects compared to developed regions (14). With the recent migration of large populations to China’s eastern coastal regions, increased housing demand and pressure to purchase homes in these areas may have affected daily life and health (47).

Regarding healthcare services, this study finds that the positive impact of healthcare technicians on LE was less pronounced in the central part of the YREB, possibly due to limitations in primary healthcare services. Over time, the overall positive impact of primary healthcare services on LE had been declining. This trend aligns with findings from the Yellow River basin in China (38). This decline may be attributed to the relatively limited positive impact of primary healthcare capacity as medical technology advances and health issues become more diversified. Consequently, improving health increasingly depends on the quality and specialization of medical services (30).

Additionally, this study explored the differences in the impact of SDOH across different types of county-level administrative divisions. In terms of demographic factors, we found that the GR had a negative impact on LE across all four types of counties, suggesting that women generally have higher LE than men in both urban and rural areas (41). Regarding FS, the negative impact of FS gradually weakened over time across all four types of counties, and by 2020, FS in autonomous counties exhibited a positive effect. This indicated that the negative health impact of larger FS had eased, though the reasons for this phenomenon require further investigation. In terms of socioeconomic factors, the LE of autonomous counties was more significantly positively influenced by education and PHA compared to the other three types of counties in the early stages of the study. In the later stages, the positive impact of income on LE in autonomous counties became more pronounced compared to the other three types. Notably, most autonomous counties are located in the western region of the YREB, so the characteristics of impact of SDOH on LE in these general counties align with those in the western region. Additionally, the influence of PHP in municipal districts, county-level cities, and counties gradually diminished over time, which may be related to the limitations of primary healthcare services in improving population health.

### 4.3 Policy implications

Improving population health and promoting spatial equity have consistently been prioritized in local public health policies. To this end, the “Healthy China 2030” Planning Outline emphasized addressing a wide range of health determinants to ensure that socioeconomic development meets the health needs of the population (26). This study revealed that the spatiotemporal patterns of LE in counties along the YREB are influenced by the differential impact of various social determinants over time and space. To promote spatial equity of population health in the region, local governments need to implement tailored and orderly policies. Specifically, from a regional perspective, it is crucial to enhance economic development in the central and western counties of the YREB, improve the employment environment, facilitate local urbanization, and strengthen local education and healthcare facilities. In the eastern counties, it is essential to manage population inflow appropriately, address the housing needs and employment conditions of local residents, and systematically guide resources such as education and healthcare services toward the central and western counties to achieve equitable allocation of public resources across regions. From an urban–rural perspective, it is important to strengthen economic development in general counties and autonomous counties, ensuring the supply of public resources such as education, healthcare, and housing. Additionally, public health resources in municipal districts and county-level cities should be redirected to support general counties and autonomous counties, promoting the equalization of urban and rural public services and reducing health disparities. Additionally, society and local governments should address the needs of disadvantaged families and improve social security policies, particularly in rural and ethnic minority areas. There should also be a broad focus on gender equality in health.

### 4.4 Strengths and limitations

This study has two strengths. First, it provided an in-depth examination of the spatiotemporal dynamics of LE at the county level, within the context of China’s cross-regional national strategic areas. This expands both the spatial scale and the study area of LE compared to previous research. Second, the paper analyzed the impact of SDOH from the perspective of spatiotemporal heterogeneity, providing insights into the variation of impacts across different types of county-level administrative divisions. This approach broadens the analytical perspectives on SDOH in existing studies.

This study also has several limitations. First, due to incomplete public health statistics at the county level in China, the selection of indicators reflecting social determinants in this paper was somewhat limited. Notably, the indicator for healthcare services relies solely on the number of PHP. Future research should focus on expanding and improving the relevant indicator system. Second, the county-level LE data used in this study comes from the national census, which is conducted every 10 years. Due to limitations in county-level statistics from earlier years, the time periods analyzed in this paper were restricted to 2000, 2010, and 2020. It is recommended that future studies incorporate more data from additional years. Third, this paper primarily analyzed the relationship between LE and SDOH from the perspective of spatiotemporal heterogeneity, which can offer valuable insights into the equitable allocation of regional public resources. However, this approach may overlook the complex interactions between various SDOH and the underlying mechanisms affecting LE. The complexity and diversity of factors affecting health make the mechanisms influencing LE neither simple nor straightforward. Therefore, future research should focus on in-depth analyses of the intrinsic relationships between different SDOH and the underlying mechanisms through which they affect LE. This approach will contribute to a deeper understanding of the relationship between health and SDOH, supporting local socioeconomic development and the equitable allocation of public resources.

## 5 Conclusion

The study revealed the spatiotemporal patterns of county LE in the YREB from 2000 to 2020. Utilizing the GTWR model, it examined the spatiotemporal variations in the impact of SDOH on LE and analyzed the differences in this influence among various county-level administrative divisions. The results indicate that from 2000 to 2020, the average LE in counties of the YREB had gradually increased, with an overall spatial pattern characterized by higher values in the east and lower values in the west. The high-high clusters were primarily concentrated in the urban agglomeration regions, while low-low clusters were predominantly located in the western region of the YREB. Among the SDOH, AYE had the strongest explanatory power for LE. The GR and FS had negative effects on LE in most counties of the YREB, while the AYE, PDI(ln), PHA, and PHP primarily had positive impacts on LE. The effects of various SDOH on LE exhibited distinct spatiotemporal heterogeneity. Furthermore, the influence of SDOH varied across different types of county-level administrative divisions and changed over time. In the future, local governments should consider relevant factors and formulate public health policies tailored to local conditions, implementing them in an orderly manner to promote spatial equity in population health.

## Data Availability

The datasets presented in this article are not readily available because the data for this study involves management restrictions from local departments. Requests to access the datasets should be directed to https://www.stats.gov.cn/sj/pcsj/.

## Footnotes

^1^ http://www.resdc.cn
